# Associations between Children’s Health-Related Characteristics, Self-Esteem, and Fathers’ Parenting Practices and Media Addiction among Younger School-Aged Korean Children

**DOI:** 10.3390/ijerph19137773

**Published:** 2022-06-24

**Authors:** Yong-Sook Eo, Yeon-Hee Lee

**Affiliations:** 1 College of Nursing, Dongguk University, Gyeongju 38066, Korea; nursingeo@dongguk.ac.kr; 2 Department of Nursing, Dong-Eui University, Busan 47340, Korea

**Keywords:** media addiction, body mass index, self-esteem, parenting practices

## Abstract

This study aimed to investigate the relationship between media addiction levels among early school-aged children and their health status, self-esteem, and their fathers’ parenting practices. Therefore, we analyzed the data from a total of 1149 fathers and children from the 10th year (2017) survey of the Panel Study on Korean Children (PSKC), by the Korean Children’s Panel Research Institute. Specifically, a multinomial logistic analysis was conducted to identify the factors affecting children’s media addiction. The media addiction levels were 68.8%, 24.9%, and 6.3%, in general, high-risk, and potential-risk users, respectively. When media addiction levels were used as the reference group for general users, the pattern observed in the data revealed a direct positive association between media use time and the probability of becoming a potential-risk user (79.4%, OR = 1.79, 95% CI: 1.20, 2.68). Furthermore, when general users were used as the reference group, the male gender accounted for the majority of high-risk users, at 99% (OR = 1.99, 95% CI: 1.46, 2.71). Within this group, the children’s body mass index (BMI) was high (5%, OR = 1.05, 95% CI: 1.00, 1.11), their media use time increased by 1 h a day (145%, OR = 2.45, 95% CI: 1.93, 3.11), their self-esteem decreased (32%, OR = 0.68, 95% CI: 0.49, 0.95), the fathers’ authoritative parenting practices were low (37%, OR = 0.63, 95% CI: 0.43, 0.92), and the permissive parenting practices were high (92%, OR = 1.92, 95% CI: 1.09, 3.37). Therefore, the results of this study highlight children’s media use time and the risk factors related to high BMIs in order to prevent media addiction among early school-aged children. Our findings also suggest appropriate parenting practices and highlight the need to strengthen children’s self-esteem.

## 1. Introduction

A wide scholarly consensus shows that today’s children are a generation that are growing up in a digital environment, which is characterized by the extensive use of computers, the Internet, and mobile phones [1]. In the last two years, this trend has been considerably accelerated by the COVID-19 pandemic, when face-to-face activities were restricted and interaction with friends decreased, while media use further increased because of distance learning [2]. Based on several recent estimates, the prevalence of children with media-use problems varies from 5% [3] to approximately 50% [4]. In South Korea, the penetration rates of smartphones and PCs are high, which has led to a considerable proportion of smartphone and PC use in a wide range of life areas, such as schedule management, social networking, and leisure activities [5]. For instance, in the group of children aged 3–9 years, Internet use accounts for a high proportion (91.2%) and among teenagers this proportion reaches 99.9% [6]. Furthermore, evidence shows that approximately one-third of all teenagers in South Korea have developed a high dependence on (are addicted to) smartphones [7]. As the age when children start using media devices such as smartphones is gradually becoming lower, the risk of media device addiction among children is showing a rapid increase, reaching 31% [8].

Media addiction is broadly defined as an individual’s psychological dependence on media devices, manifesting itself in the extensive and prolonged use of media devices. Media addiction has been reported to be associated with difficulties in individuals’ overall life, such as feeling anxious when not using media devices [9]. School-aged children who are exposed to media devices from an early age have weak self-control over their use of devices such as mobile phones and develop a high sensitivity to media. This poses a great risk that these children will develop a media addiction and that they will experience the corresponding negative effects that this can have on this age group [10,11].

Overall, extensive evidence shows that media addiction negatively affects individuals’ health, which can result in physical [12] and emotional problems such as depression and anxiety [1,13]. Furthermore, media addiction decreases children’s likelihood of engaging in healthy interactions with their peers and families [14] and leads to behavioral problems such as impulsive behavior [15] and poor adaptation to school life [16]. Furthermore, as young children become accustomed to stimulating digital media they lose interest in conventional media that offer less stimulation, such as books, which, in turn, leads to delays in children’s cognitive development [17]. In general, children’s vulnerability to media addiction can be related to difficulties in self-regulation [18] and an immature ability to control themselves [19], which are typical of this age group. Therefore, in the present-day context, where the risk of addiction increases because of the early age of children’s first media exposure and use, the importance of early media addiction prevention among early school-aged children can hardly be overestimated.

Previous studies have explored some factors that lead to problematic media use in children [20]. Among these factors, scholars differentiate between personal factors, such as the children’s gender, age, and personality, and environmental factors, such as family and school. In terms of personal factors, there have been several studies on children’s self-control and self-esteem [20,21]. Self-esteem is broadly defined as the recognition of oneself as a valuable person and a positive evaluation of one’s own perception [22]. Children with low self-esteem have lower adaptability to the environment and are more easily exposed to problematic or deviant behaviors [23], thus, such children develop a strong tendency to over-immerse themselves in the media [24]. Notably, low self-esteem was reported to be a major factor involved in problematic media use among late school-aged children [25] and adolescents [26], and young adults [27]. For instance, several previous studies observed a strong correlation between self-esteem and the level of smartphone addiction [20,21]; however, most of these studies targeted late school-aged and adolescent children.

Other factors that may be related to children’s problematic media use have not been investigated thoroughly. Little is known about the link between children’s health-related factors and media addiction. For instance, one relevant study reported a strong link between children’s health-related characteristics, such as obesity and sleep disorders, and Internet addiction [28]. However, considering the scarcity of relevant research, it is necessary to further investigate the relationship between children’s self-esteem and health-related characteristics, and media addiction, with a specific focus on early school-aged children.

In other previous studies, among the environmental factors, family had a different relationship with children’s media addiction based on the children’s age, and it was found to be a major influencing factor at a relatively young age [29]. Parenting practices differed in relation to children’s media addiction based on their patterns, for instance, a coercive (compulsive) attitude from the parent increased the children’s media dependence, while an autonomous attitude from the parent lowered it [15]. In addition, the rate of smartphone addiction among school-aged children was lower for children whose parents displayed a more autonomous attitude to their use of technology, than it was for the children whose parents applied hard parenting practices to their children’s smartphone use [30]. Thus, the results of these studies show that the parenting practice patterns employed by the parents of children (up to late-school age) can play an important role in children’s development of media addiction. However, one limitation of most of these previous studies is that they focused on mothers’ parenting practices in relation to children’s media addiction, with very few studies considering the impact of fathers’ parenting practices. In addition, interactions with their peers and the school community were also reported as relevant factors [10]. As media addiction in children has been seen to differ depending on their age, a comprehensive approach, which includes the interaction of personal and environmental factors, should be considered.

Based on the ecological systems theory [31], this study intended to analyze the influencing factors of media addiction on the interaction between early school-aged children and their surrounding environment. Factors related to age differ in the literature on smartphone addiction [29], and most factors produce contradictory findings [20]. This suggests that it is necessary to explore the relevant predictors in the interaction between children and their environment.

To fill this gap, in the present study we investigated the relationship between the media addiction levels among early school-aged children, and personal (i.e., the children’s health status and self-esteem) and environmental (i.e., fathers’ parenting practices) factors. The specific research questions addressed in this study were as follows: (1) What is the level of media addiction in early school-aged children? (2) Are children’s health status, self-esteem, and fathers’ parenting practices related to children’s media addiction? 

The remainder of this study paper is structured as follows: section two presents the methodology used in the present study; the results are reported in section three; in sections four and five, we discuss the findings and limitations of the study; and section six concludes the study.

## 2. Materials and Methods

### 2.1. Participants and Ethical Consideration

This was a descriptive study that aimed to identify the factors related to media addiction among early school-aged children. For the analysis, we used the data from the 10th year of the Korean Children’s Panel Study (PSKC), conducted in 2017 by the Korean Institute of Child Care and Education (KICCE) [32]. The Korean Children’s Panel Survey is a panel study that tracks newborns born in 2008 at medical institutions sampled nationwide, until 2027 (KICCE, 2016). First-year data were collected from a total of 2150 newborns, born between April and July 2008, and the survey has been conducted every year thereafter. Data for 2017, i.e., the 10th year, include the data of 1484 parents and their nine-year-old children. Data for the analysis in the present study were obtained after submitting the researcher’s affiliation and the purpose of the data’s use to the Korean Child Rearing Policy Research Institute. The participants’ personal, identifiable information was deleted (http://panel.kicce.re.kr (accessed on 3 January 2022)). Only the records without missing values in the main variables analyzed in this study—namely, children’s health-related characteristics, self-esteem, and fathers’ parenting practices—were selected for further analysis. After filtering incomplete records, the final sample contained a total of 1149 people. All participants provided their informed consent to the PSKC survey team before their inclusion in the survey (IRB No. KICCEIRB-2017-No.05).

### 2.2. Instruments

Demographic characteristics of children and fathers, including children’s gender, age and fathers’ age, education level, and economic status were selected to understand the predictors of media addiction among early school-aged children. The media-use-related characteristics of children included whether or not they possessed a smartphone and the daily use of media, such as smartphones, PCs, and the Internet.

For media addiction, a modified version of the Internet addiction observer tool (KO scale), developed by the Internet Addiction Response Center (iapc.or.kr) of the National Information Society Agency, was used [33]. In the revised tool, references to the Internet were changed to PC/smartphone. Our assumption was that all activities such as games, social network services (SNS), video viewing, and Internet use using PCs/smartphones were indicative of media addiction. Mothers were allowed to observe and measure their children’s behavior. The survey consisted of 15 items in the following four categories: (1) five items related to obstacles in daily life, (2) four items related to withdrawal, (3) four items related to tolerance, and (4) two unclassified items. Each item was evaluated on a 4-point Likert scale, with higher scores indicating a higher likelihood of addiction. With regard to media addiction, based on the total score and the total score in each category, all users were classified in the following three groups. First, general users were the participants who scored 12 points or less for daily-life obstacles, 10 points or less for withdrawal items, 9 points or less for tolerance items, and with a total score of 27 points or less. Second, potential-risk users would rate 28 to 29 points or less in total, 13 points or more for items related to daily-life obstacles, 11 points or more for withdrawal items, and 10 points or more for tolerance items. The third group embraced high-risk users with a total score of 30 or higher, or who met the following criteria: 14 points or higher for obstacles in daily life, 12 points or higher for withdrawal items, and 11 points or higher for tolerance items. In this study, Cronbach’s α was 850.

For the health status of children, we evaluated body mass index (BMI), sleep time, and subjective health status. BMI was calculated based on the data concerning children’s weight and height, as measured by a public health teacher in school. For sleep time, the average daily sleep time of children was computed by adding up the weekday and weekend hours. The children’s subjective health status was assessed on a 5-point scale, based on the health status of each child as evaluated by the mother, with scores ranging from 1 (very unhealthy) to 5 (very healthy). Accordingly, higher scores indicated better health status.

To measure self-esteem, we used a modified version of Rosenberg’s [22] self-esteem scale of 10 items. These were reduced to five items because of the age of the children, and the phrasing of the items was modified to ensure the respondents fully understood the meaning of the items. The self-esteem survey was rated on a 4-point scale, ranging from 1 (not at all) to 4 (strongly agree). Accordingly, a higher score meant higher self-esteem. Cronbach’s α in the study was 0.754.

For fathers’ parenting practices, we used the parenting styles and dimensions questionnaire (PSDQ) that was developed by Robinson et al. [34] and translated and modified by the Korean Children’s Panel researchers. The parenting practices scale consisted of a total of 62 items in the following 3 categories: (1) authoritative (27 items), (2) authoritarian (20 items), and (3) permissive (15 items). The items were rated on a 5-point scale, ranging from 1 (not at all) to 5 (strongly agree). Cronbach’s α values for the authoritative, authoritarian, and permissive parenting styles were 0.909, 0.890, and 0.986, respectively.

### 2.3. Procedure

The Korean child growth and development longitudinal study (Korean Children’s Panel II) is a study that tracks children’s growth and development over their first 20 years of life, from birth to adulthood. This study was launched with a panel of 2,150 newborns, born in 2008. In 2017, the data were collected for the 10th year, when children were in the third grade of elementary school. Excluding 380 households from the rejection panel, we analyzed a total of 1,685 households. The participants were children, parents, and teachers. First, basic information such as confirmation of the visiting address was checked using a telephone survey, and a paper-based survey questionnaire was sent out before the researcher’s visit.

Subsequently, we conducted an interview, observational survey, and performance test. These were accomplished through personal interviews using a computer-programmed questionnaire that could transfer the questionnaire results to a tablet. For the analysis of the variables in the present study, we focused on children’s media addiction and health status, as evaluated by their mothers; the fathers’ parenting practices; and self-esteem, as evaluated by children. Before the data survey, we conducted questionnaire preparation, Institutional Review Board (IRB) deliberation, computer program construction, and researcher training. Given that researchers had to collect multi-faceted data from a variety of participants, including children, parents, and teachers, they were trained through pre-education, which included lecture-style explanations, mock exercises, questions and answers, and comprehension tests. Data collection was conducted from July to November 2017, and the final data of 1,484 households were collected.

### 2.4. Data Analysis

The data were statistically analyzed using SPSS 25.0 program (IBM Corp., Armonk, NY, USA). Frequency analysis and descriptive statistics were conducted to analyze the general characteristics of the participants, children’s health-related characteristics, media addiction levels, self-esteem, and fathers’ parenting practices. The differences between the participants’ general characteristics, health-related characteristics, and media addiction levels were evaluated using chi-square test. In order to analyze the effect of the main variables on the level of media addiction, multinomial logistic regression analysis was performed, where general users were classified as the reference group, and potential-risk and high-risk were classified as the comparison group.

## 3. Results

### 3.1. Characteristics of Children and their Fathers

Regarding the gender of the children included in our sample, male students accounted for 51.6%. In terms of their order of birth in their respective families, 46.7% were firstborns. The average age of the fathers was 42.15 years, with an average income of KRW 5,355,600 per month, with 60.9% of the fathers above the median. Furthermore, 31.3% of the children had a smartphone, and their media use time amounted to 1.37 h a day. Most children (86.4%) used a smartphone for less than 3 h a day, but 5.9% used it for more than 3 h a day (see Table 1).

### 3.2. Children’s Media Addiction Levels, Health-Related Characteristics, Self-Esteem, and Fathers’ Parenting Practices

The average level of smartphone addiction among the participants was 25.1 point, the highest among the general (68.8%) and high-risk users (24.9%). For health-related characteristics, the average BMI of the children was 17.95, the average sleep time was 9.44 h per day, and the subjective health status was 4.27 points. Self-esteem was 17.42 out of 25; and fathers’ parenting practices was authoritative 99.19 (out of 135), authoritarian 45.76 (out of 100), and permissive 38.45 (out of 75) (see Table 2).

### 3.3. Differences in Media Addiction Levels Based on the General Characteristics

Media addiction levels among the participants significantly varied depending on the children’s gender (χ2 = 29.34, *p* < 0.001), smartphone possession (χ2 = 5.97, *p* = 0.050), and media use time (χ2 = 36.92, *p* < 0.001). In the case of boys who did not own a smartphone, there were more high-risk users in the group with less than 3 h of media use per day (see Table 3).

### 3.4. Predictors of Children’s Media Addiction Level

Table 4 summarizes the results of the predictors of media use addiction among younger school-age Korean children. First, the results revealed that the influence of each variable on potential-risk users’ level of media addiction, using general users as the reference group, and their media use time (B = 0.59, *p* = 0.004) was significant. In other words, the longer the children’s media use time, the higher their probability of becoming potential-risk users, compared to that of the general users (79.4%, OR = 1.79, 95% CI: 1.20, 2.68). Second, with regard to the influence of each variable on the media addiction level and the media use level for high-risk users, compared to that of the general users, the significant influencing factors were gender (B = 0.69, *p* < 0.001), BMI (B = 0.05, *p* = 0.035), media use time (B = 0.90, *p* < 0.001), self-esteem (B = −0.39, *p* = 0.022), authoritative parenting (B = −0.47, *p* = 0.016), and permissive parenting practices (B = 0.65, *p* = 0.024). That is, the probability of becoming a high-risk user was found to be 99% more likely for boys than for girls (OR = 1.99, 95% CI: 1.46, 2.71), and 5% more likely for those with a high BMI (OR = 1.05, 95% CI: 1.00, 1.11). Furthermore, a one-hour per day increase in media use time increased the probability of becoming a high-risk user by 145% (OR = 2.45, 95% CI: 1.93, 3.11). Furthermore, the probability of becoming a high-risk user among children with lower self-esteem was 32% (OR = 0.68, 95% CI: 0.49, 0.95). Finally, the probability of becoming a high-risk user increased when fathers’ authoritative parenting was lower (37%, OR = 0.63, 95% CI: 0.43, 0.92) and when fathers’ permissive parenting practices were higher (92%, OR = 1.92, 95% CI: 1.09, 3.37).

## 4. Discussion

This study investigated the factors affecting media addiction among early school-aged children in South Korea. The results revealed that media addiction levels were 68.8%, 24.9%, and 6.3% in general users, high-risk, and potential-risk users, respectively. In comparison with our results, a previous study with a representative sample of Korean children and adolescents found that Internet and smartphone dependence rates were 14.4% and 10.2%, respectively, suggesting that approximately 24.6% of Korean children and adolescents are at risk of developing Internet and smartphone media addiction [35]. The observed difference in the media addiction rate can be related to the difference in the manipulation of media addiction. In the present study, issues such as daily life disorders, withdrawal, and a high tolerance of all typical media activities, such as games, SNS, video viewing, and Internet access, were considered forms of media addiction. While earlier studies on the use of media devices predominantly focused on Internet addiction in relation to using a PC [36], after 2011, with the increase in smartphone penetration, several studies addressed smartphone addiction [37] and problematic smartphone use [13]. More recently, there have been studies on the phenomenon of “phubbing”, which refers to the practice of ignoring one’s surroundings while looking at a smartphone [38].

Another reason underlying the difference in media addiction rates that was observed in the present and previous studies is related to the difference in how media addiction is evaluated by the observers, such as experts, teachers, parents, and children’s self-reports. In a previous study that compared these two types of evaluation, smartphone addiction, as perceived by the children themselves, was 16.4%, whereas smartphone addiction, as perceived by their parents, was 47.5% [2]. A limitation associated with children’s self-reporting is denial, as most children come to the counseling room accompanied by their parents, which tends to reduce the addiction situation [36]. In contrast, if the observers are parents, the overall information and education on media addiction are based on a more objective scale. In the present study, we relied on the media addiction data observed by mothers, who are the primary caregivers of children. Our results suggest an urgent need to prepare adequate guidelines for parent education, which inform caregivers of the problems associated with children’s media use and of the need to teach children to use media appropriately in order to help them grow and develop. In particular, with the outbreak of the COVID-19 pandemic, smartphone use and abuse and addiction among children have significantly increased [37]. Therefore, parents must understand their children’s media addiction symptoms and teach them to use media effectively and safely.

When media addiction levels were analyzed for general users (as the reference group) it was found that the longer their media use time, the higher the probability of a child becoming a potential-risk user. Consistently with these findings, in a previous study on the factors of smartphone addiction among preschool-aged children, smartphone addiction was found to increase with an increase in the time spent using smartphones [39]. Specifically, the authors of the aforementioned study found that in families where mothers were more permissive with regard to their children’s smartphone use (i.e., the perceived mother’s permission level) children were more likely to become addicted to their smartphones.

Our findings on the direct relationship between media use time and media addiction are in line with previous studies on young [17] and late school-aged children (fifth and sixth grade of elementary school) [40]. In our data, 31.3% of the participants had a smartphone, and the average media use time per day amounted to 1.37 h. Based on a longitudinal study that examined the relationship between changes in media use time and media addiction among early school-aged children [16], children whose media use time continuously increased showed a strong tendency toward media addiction, as did those who spent more time using media at an early age. Therefore, these findings show that the risk of the development of media addiction is higher in children who are first exposed to media earlier and who have a significantly increased media use time. From an applied perspective, these findings underscore the importance of managing media use time at a young age.

Furthermore, we found a strong relationship between media addiction and children’s gender, with boys being more likely to become high-risk users. A similar conclusion was drawn in a previous study on children of the same age [41] and students [42,43]. In contrast, however, other studies reported that girls are at a high risk of smartphone addiction [44]. For instance, in a study targeting adolescents, the smartphone addiction group consisted of 63.6% female students and 36.4% of male students [45]. Considering the lack of consistency in previous studies, further studies on the impact of age and gender on media addiction are required.

Another finding of the present study is the relationship between BMI and media addiction. Specifically, in our results, participants with a high BMI had a higher probability of becoming a high-risk media user, with their risk increasing by 5%. In line with this finding, a previous study on the relationship between Internet addiction and health-related characteristics, such as obesity and sleep disorders, revealed that BMI was associated with Internet addiction in Korean and American children, but not with sleep disorders [46]. Notably, most studies examining the relationship between media addiction and BMI perceived BMI as an outcome variable of media addiction [28,47]. There is extensive evidence that an increase in media use decreases the amount of physical activity, which, in turn, leads to obesity. Regarding childhood obesity, an important issue to note is that childhood obesity is a risk factor for psychosocial problems such as bullying, social stigma, and peer rejection [48]. Therefore, considering that psychological atrophy and activity restriction can aggravate media addiction, BMI needs to be carefully considered as a predictor of media addiction in children.

Another important predictor of media addiction observed in our results was children’s self-esteem. Here, we observed that the lower the child’s self-esteem, the higher the probability of their becoming a high-risk user. Our results on this factor agree with previous reports that self-esteem is a major influencing factor for media addiction in late school-aged children [25], particularly with regard to smartphone use [1]. Overall, low self-esteem is a characteristic of an addictive personality [49] and there is evidence that individuals who do not value themselves easily experience peer pressure and engage in addictive behaviors [50]. The results of our study reveal that low self-esteem is a strong predictor of media addiction, even in the early years of school. Accordingly, a child’s personal characteristics can act as a protective factor in preventing media addiction. As concluded in a literature review on problematic media use by children and adolescents, self-esteem and self-control are individual factors [20]. Furthermore, a longitudinal study that examined the temporal effects of media use time and media addiction found no difference between media use time and age [16], indicating that it is important for children to manage and control their media use time individually. Therefore, our previous findings suggest that preventing media addiction involves strengthening personal factors such as children’s self-esteem.

In this study, personal factors were found to be important predictors of media addiction in early school-age children. As media addiction relates to an individual’s daily life [51], time management and control interventions are needed, as well as interventions that strengthen self-esteem. In addition, it is necessary to examine the daily activities and psychological atrophy of children in the high-risk group, especially boys with a high BMI.

Finally, we also observed that fathers’ authoritative and permissive parenting practices are strongly related to media addiction. More specifically, we found that less authoritative and more permissive parenting practices were associated with a higher probability of children becoming high-risk users. Authoritative parenting practices are also referred to as democratic parenting practices, as they use gentle controlling methods [52]. Although a parent is the supreme authority and wants their child to obey the rules, an authoritative parent exhibits good parenting practices, which recognize that children have an independent personality and different opinions. Subsequently, permissive parenting practices are inconsistent with the rules and discipline standards and provide minimal control over the children’s behavior [52], therefore, they can be viewed as undesirable parenting practices. Even in late school-aged children, strong parental control over smartphone use was found to be a major influencing factor for smartphone addiction [30], which is in line with our findings that parental guidance on media use mediates media addiction tendencies and positively affects children’s language development [17,30]. To generalize, our findings suggest that parents showing strong control over school-aged children’s undesirable behaviors, whilst also respecting their children’s personalities, is an important factor that prevents media addiction. Moreover, our results also revealed that permissive parenting practices aggravated smartphone addiction [29]. However, in contrast to previous studies, which showed that coercive parental practices increase dependence on smartphones [15], we did not find any evidence that authoritarian parenting practices that use coercive control methods lead to the development of media addiction. Furthermore, in a previous study on fourth-grade elementary (10 years old) and first- grade middle school students (13 years old), parenting practices were reported to be related to smartphone addiction in the fourth-grade elementary students, but not in the first-grade middle school students, and parental factors were not related to developmental age. An important contribution of the present study is that we focused on fathers’ parental practices, rather than mothers’. Considering the growing participation of fathers in child rearing, their role has become more important [53], and several studies have already underscored the role of fathers in their children’s development of media addiction [54,55]. Compared with general users, children who were addicted to media use perceived social support (e.g., understanding, interest, information provision, and advice from their fathers) to be weaker [54]. Furthermore, a qualitative study of fathers’ experiences with Internet addiction found that fathers tend to show a lack of understanding of the appropriate use of the Internet and are more addicted to media than their children. This highlights the need for proper education on adequate media use [54]. Overall, the relationship between children’s Internet addiction and their fathers’ roles in their development requires further study. In addition, this study only explored the behavior of parents as environmental influencing factors, but further research would benefit from exploring the relationship between same-age peers and the influence of teachers on media addiction in early school-age children.

## 5. Limitations of the Study

There were several limitations of this study that should be considered when interpreting these findings. Firstly, in the sample selection the target group was formed by applying a multi-stage and proportional sampling method, and external validity was secured in the 10th sample size. However, it was difficult to ascertain detailed information about the media use of children and the related factors from this. A better interpretation would have been possible if other elements, such as physical activity and nutritional intake, were included. Secondly, this study was limited in its ability to establish a causal relationship between the variables, as the data used came from a cross-sectional sample. Future studies should use longitudinal panel data to examine media addiction trends and the related factors in children in the early, middle, and late stages of school.

## 6. Conclusions

The results of this study showed that self-esteem is a significant predictor of media addiction among early school-aged children. Therefore, to effectively prevent the development of media addiction in this age group, it is necessary to strengthen children’s self-esteem so that they develop a sense of control over their media use. Furthermore, considering our finding that BMI is an important factor that predisposes individuals to media addiction, it is necessary to examine whether the psychological atrophy and distorted body image caused by obesity can adversely affect self-esteem. Furthermore, we found that children’s media addiction was predicted by fathers’ use of authoritative and permissive practices. Thus, our results suggest that the children’s media use time, which we found to be the most powerful factor in media addiction, should be appropriately controlled by their parents, but that this should also be combined with parents’ efforts to maintain their relationships with their children in a gentle manner.

This study fills the gap in the literature regarding the relationship between media addiction among early school-aged children, and their health-related characteristics and parenting practices. In addition, this study provides basic data for community health providers and schoolteachers in their elaboration of effective prevention programs regarding media addiction among early school-aged children.

## Figures and Tables

**Table 1:** Participants’ characteristics (N = 1149).

Characteristics	Categories	*n* (%) or M ± SD
General Characteristics of Children and Their Fathers		
Gender	Male	593 (51.6)
	Female	556 (48.4)
Birth order	First	537 (46.7)
	Second	480 (41.8)
	Third and above	132 (11.5)
		42.15 ± 3.83
Fathers’ age (years)	≤39.9	283 (24.6)
	40.0–44.9	583 (50.7)
	≥45.0	283 (24.6)
Fathers’ education level	≤High school graduate	299 (26.0)
	≥University graduate	850 (74.0)
		5,355,600 ± 450,450
Economic level	Very low (≤3,400,000)	208 (18.1)
(KRW/month)	Low (3,400,001–4,000,000)	241 (21.0)
	Moderate (4,000,001–5,300,000)	316 (27.5)
	High (≥5,300,001)	384 (33.4)
Media use characteristics of children		
Smartphone	Have	360 (31.3)
	Have not	789 (68.7)
		1.37 ± 0.59
Media use time(hours)	No	89 (7.7)
	- < 1	236 (20.5)
	1- <2	522 (45.4)
	2- < 3	235 (20.5)
	≥3	67 (5.9)

M—mean; SD—standard deviation.

**Table 2:** Children’s media device addiction levels, self-esteem, and fathers’ parenting practices (N = 1149).

Characteristics (Items)	*n* (%) or M ± SD	Min	Max
Media device addiction (15)	25.10 ± 6.07	15.00	51.00
General users: ≤27	790 (68.8)		
Potential risk users: 28–29	73 (6.3)		
High risk users: ≥30	286 (24.9)		
Health-related characteristics of children			
BMI (kg/m^2^) (percentile)	17.95 ± 2.94	11.81	29.76
Sleep time (hours)	9.44 ± 0.65	6.50	12.50
Subjective health status (points)	4.27 ± 0.75	1.00	5.00
Children’s self-esteem (5)	17.42 ± 2.16	5.00	20.00
Fathers’ parenting practices			
Authoritative (27)	99.19 ± 11.98	59.00	132.00
Authoritarian (20)	45.76 ± 9.83	23.00	77.00
Permissive (15)	38.45 ± 4.60	21.00	56.00

M—mean; max—maximum; min—minimum; SD—standard deviation.

**Table 3:** Differences in media device addiction based on participants’ characteristics (N = 1149).

Characteristics	Categories	Media Addiction			χ^2^ (*p*)
		General Users (*n* = 943)	Potential-Risk Users (*n* = 94)	High-Risk Users (*n* = 352)	
Gender	Male	367 (46.5)	40 (54.8)	186 (65.0)	29.34 (<0.001)
	Female	423 (53.5)	33 (45.2)	100 (35.0)	
Birth order	First	375 (47.5)	37 (50.7)	125 (43.7)	2.95 (0.566)
	Second	328 (41.5)	30 (41.1)	122 (42.7)	
	Third and above	87 (11.0)	6 (8.2)	39 (13.6)	
Fathers’ age (years)	≤39.9	188 (23.8)	22 (30.1)	73 (25.5)	2.85 (0.582)
	40.0- < 45.0	404 (51.1)	38 (52.1)	141 (49.3)	
	≥45	198 (25.1)	13 (17.8)	72 (25.2)	
Fathers’ educational level	≤High school graduate	196 (24.8)	17 (23.3)	86 (30.1)	3.32 (0.190)
	≥University graduate	594 (75.2)	56 (76.7)	200 (69.9)	
Economic level(KRW/month)	Very low (≤3,400,000)	129 (16.3)	15 (20.5)	64 (22.4)	9.34 (0.156)
	Low 3,400,001–4,000,000)	162 (20.5)	13 (17.8)	66 (23.1)	
	Moderate (4,000,001–5,300,000)	230 (29.1)	17 (23.3)	69 (24.1)	
	High (≥5,300,001)	269 (34.1)	28 (38.4)	87 (30.4)	
Smartphone	Have	263 (33.3)	24 (32.9)	73 (25.5)	5.97 (0.050)
	Have not	527 (66.7)	49 (67.1)	213 (74.5)	
Media use time(hours)	No	76 (9.6)	2 (2.7)	11 (3.8)	92.308 (<0.001)
	- < 1	191 (24.2)	9 (12.3)	36 (12.6)	
	1- < 2	373 (47.2)	39 (53.4)	110 (38.5)	
	2- < 3	123 (15.6)	15 (20.5)	97 (33.9)	
	≥3	27 (3.4)	8 (11.0)	32 (11.2)	

M—mean; SD—standard deviation.

**Table 4:** Multinomial logistic regression for predictors of media device addiction among younger school-aged children.

Variables	Media Device Addiction							
	General vs. Potential-Risk Users				General vs. High-Risk Users			
	B	SE	*p*	OR (95% CI)	B	SE	*p*	OR (95% CI)
Intercept	−0.99	2.93			−2.16	1.76		
Sex (ref = female)	0.31	0.26	0.241	1.36 (0.81, 2.27)	0.69	0.16	<0.001	1.99 * (1.46, 2.71)
Sleep time (per 1 h)	−0.10	0.20	0.625	0.91 (0.62, 1.34)	−0.10	0.12	0.373	0.90 (0.72, 1.13)
BMI (per 1 point)	0.02	0.04	0.669	1.02 (0.94, 1.11)	0.05	0.03	0.035	1.05 * (1.00, 1.11)
Children’s perceived health status (per 1 point)	−0.01	0.17	0.964	0.99 (0.71, 1.39)	−0.05	0.10	0.659	0.96 (0.78, 1.17)
Smartphone (ref = have not)	0.02	0.28	0.954	1.02 (0.59, 1.74)	0.29	0.17	0.086	1.34 (0.96, 1.87)
Media use time (per 1 h)	0.59	0.21	0.004	1.79 * (1.20, 2.68)	0.90	0.12	<0.001	2.45 * (1.93, 3.11)
Self-esteem (per 1 point)	−0.36	0.28	0.203	0.70 (0.41, 1.21)	−0.39	0.17	0.022	0.68 * (0.49, 0.95)
Father’s parenting practices (per 1 point)								
Authoritative	−0.27	0.32	0.397	0.77 (0.41, 1.42)	−0.47	0.19	0.016	0.63 ^*^ (0.43, 0.92)
Authoritarian	0.57	0.33	0.079	1.78 (0.94, 3.37)	0.36	0.20	0.065	1.44 (0.98, 2.12)
Permissive	−0.34	0.48	0.479	0.71 (0.28, 1.81)	0.65	0.29	0.024	1.92 * (1.09, 3.37)

−2 Log likelihood = 1595.66; χ^2^ = 154.38; df = 20 Cox and Snell; R^2^ = 12.8; Nagelkerke R^2^ = 16.3. * *p* < 0.05.

## Data Availability

Data are available upon request due to ethical and privacy restrictions.
